# Person-Centered Care Environment Associated With Care Staff Outcomes in Long-Term Care Facilities

**DOI:** 10.1097/JNR.0000000000000412

**Published:** 2020-11-30

**Authors:** JiSun CHOI, Da Eun KIM, Ju Young YOON

**Affiliations:** 1 PhD, RN, Associate Professor, College of Nursing Science, Kyung Hee University, Seoul, Republic of Korea; 2 PhD, RN, Assistant Professor, College of Nursing, Research Institute of Nursing Science, Kyungpook National University, Daegu, Republic of Korea; 3 PhD, RN, Associate Professor, College of Nursing, Research Institute of Nursing Science, Seoul National University, Seoul, Republic of Korea.

**Keywords:** intent to leave, job satisfaction, nursing home, nurse staffing, person-centered care

## Abstract

**Background:**

Although a general implementation of person-centered care in Korean long-term care delivery systems would be challenging, person-centered care has the potential to improve resident and staff outcomes through changes in current care services. However, little empirical evidence currently supports a positive relationship between person-centered care environments and staff outcomes.

**Purpose:**

This study was designed to examine the relationship between person-centered care environments and staff outcomes, including job satisfaction and turnover intention, among care staff in Korean long-term care facilities.

**Methods:**

This descriptive, correlational study used data from 235 care staff (94 nursing staff and 141 personal care workers) in 13 long-term care facilities in Korea. Data were collected using structured survey questionnaires, including items related to the person-centered care environment, job satisfaction, and turnover intention. Multilevel linear and logistic regression analyses were performed using Mplus Version 7.0.

**Results:**

After controlling for individual (age, education, monthly income, position, shift work, and job tenure) and organizational (type of facility, location, ownership, bed size, and staffing levels) characteristics, a significant relationship was found between the person-centered care environment and job satisfaction and turnover intention among staff in Korean long-term care facilities.

**Conclusions/Implications for Practice:**

The study findings indicate that working in a person-centered care environment is key to higher job satisfaction, which is a significant predictor of turnover intention among staff in long-term care facilities. To recruit and retain qualified staff to provide high-quality person-centered care in long-term care facilities, a supportive work environment is crucial. Fostering a person-centered care environment will ultimately improve quality of care for residents.

## Introduction

The rapid increase in the aging population worldwide has led to a growing demand for long-term care facilities such as nursing homes and skilled nursing facilities for older adults who experience cognitive or functional impairment (McGilton et al., 2016). However, the chronic shortage of long-term care staff, including registered nurses (RNs), certified nursing assistants (CNAs), and personal care workers (PCWs), who are essential caregivers in these facilities, is a pressing concern (Bethell et al., 2018; Radford et al., 2015; Zhang et al., 2019). Research findings have shown a significant and negative association between a high rate of turnover for long-term care staff and the quality of resident care in terms of outcome variables such as frequency of nursing care deficiencies and incidence of pressure ulcers (Lerner et al., 2014; Trinkoff et al., 2013). Thus, stability in the nursing care workforce is vital to the improvement of care quality in long-term care settings.

Considering the complex care needs of residents living in long-term care facilities, it is commonly recognized that work in these facilities presents physical and emotional challenges (McGilton et al., 2016). Factors related to turnover and turnover intention among staff in long-term care settings include job satisfaction, supportive supervision, work shift, demographic characteristics (e.g., age and education), and organizational characteristics (e.g., staffing levels, bed size, and location; Karantzas et al., 2012; Zhang et al., 2019). Job dissatisfaction is one of the most cited predictors of turnover intention among long-term care staff (Choi & Johantgen, 2012; Karantzas et al., 2012; Kuo et al., 2014). Interestingly, however, several researchers have found that CNAs and care aides working in long-term care facilities, although generally satisfied with their work, are less satisfied with their working conditions, which include heavy workloads, lack of appreciation and recognition, and lack of the resources necessary to provide quality care (Chamberlain et al., 2016; Choi & Johantgen, 2012; Squires et al., 2015). Moreover, job satisfaction is a significant mediator in the relationship between work stress and turnover intention among long-term care nurses (Kuo et al., 2014).

In addition to job satisfaction, researchers have investigated work environments for long-term care staff, showing a significant relationship between a supportive work environment, job satisfaction, and lower turnover intention (Chamberlain et al., 2016; Choi & Johantgen, 2012; Gilster et al., 2018; Squires et al., 2015). A person-centered care environment consists of care delivery systems that facilitate individualized care based on a holistic approach through a therapeutic relationship (Edvardsson et al., 2009) and is crucial for job satisfaction among long-term care staff (McCormack et al., 2010). In addition, there have been great efforts to create a more person-centered care environment as a key element of quality care for residents in long-term care facilities (Edvardsson et al., 2017; McCormack et al., 2010; McCormack & McCance, 2006).

The previously discussed trends apply to Korea, which has experienced the most rapid growth in aging population in the world, with 14.3% of the population older than 65 years old in 2018 (Statistics Korea, 2018). The consequent rapid growth in new, long-term care facilities for vulnerable older adults in Korea has raised serious concerns such as nursing staff shortage, poor working conditions, and poor quality of resident care (H. Y. Lee et al., 2015, M. Lee et al., 2013). In response to the demand for higher quality care in long-term care facilities, there has been growing interest in person-centered care approaches that involve shifting the focus from caregiver tasks to individual residents (McCormack & McCance, 2006). Although developing person-centered care delivery systems in all Korean long-term facilities would be a difficult challenge, successful implementation of person-centered care into practice may improve the current state of long-term care services as well as resident and staff outcomes in Korea. In one study, a significant association was found between person-centered care and nursing care quality in Korean long-term care hospitals (Sagong & Lee, 2016). However, little empirical evidence is currently available to confirm the positive effect of person-centered care environments on staff outcomes in Korean long-term care hospitals. Therefore, this study was conducted to investigate the relationship between person-centered care environments and staff outcomes, including job satisfaction and turnover intention, among staff working in long-term care facilities in Korea.

## Methods

### Study Design

The data in this descriptive, correlational survey study were collected between November 2016 and February 2017 from a convenience sample of care staff, including nursing staff (RNs and CNAs) and PCWs, who had worked for more than 1 month in their current facility.

### Study Sample and Data Collection

In long-term care facilities in Korea, both RNs and CNAs are considered nursing staff and provide nursing care to residents. PCWs assist residents with most direct care and daily activities such as feeding, bathing, toileting, and other personal needs. The parent study identified the overall relationships among person-centered care and care outcomes from the perspectives of “residents” and “staff” and conducted psychometric testing of person-centered care instruments in a Korean cultural setting. This study, part of the parent study, focused on person-centered environment and staff outcomes. For this study, the minimum sample size of 150 was calculated based on the rule of thumb for regression analysis of at least 10 cases per estimated parameter (Waltz et al., 2017), with 15 predictors included in our regression models. The final analytical sample consisted of 235 staff, including 94 nursing staff and 141 PCWs in 13 long-term care facilities in Korea (IRB No. E1811/001-001).

### Measures

#### Staff outcomes

The two staff outcome variables considered in this study were job satisfaction and turnover intention. Job satisfaction was measured using the job satisfaction subscale of the Korean version of the Copenhagen Psychological Questionnaire II (COPSOQ II-K; June & Choi, 2013). The COPSOQ II was originally developed by the National Institute of Occupational Health Research in Denmark as a comprehensive measure of the psychosocial working environment (Pejtersen et al., 2010). In the COPSOQ-K, the job satisfaction subscale consists of four items that are scored on a 4-point Likert scale, with higher scores indicating higher job satisfaction. Good reliability and validity were established for the subscale, with a Cronbach's alpha of .78, and concurrent validity, using correlation analysis with Korean job stress, was assessed as −.67 in a psychometric testing study of the COPSOQ-K (June & Choi, 2013). The Cronbach's alpha was .88 in this study. Turnover intention was measured using a single, dichotomously scored item (1 = *yes*, 0 = *no*), asking whether the respondent planned to leave within 1 year.

#### Person-centered care environment

Person-centered care environment was measured using the Korean version of the Person-centered Climate Questionnaire-Staff version (PCQ-S), translated by Sagong et al. (2018). The PCQ-S was originally developed in Sweden by Edvardsson and colleagues to measure the care environment as perceived by staff in healthcare settings in which person-centered care is provided (Edvardsson et al., 2009). The PCQ-S comprises 14 items in three subdomains: “climate of safety,” “climate of everydayness,” and “climate of community.” The items are rated on a 6-point Likert scale, with higher scores indicating a more person-centered care environment. The reliability and validity of the Korean version of the PCQ-S were established, with strong evidence of internal consistency reliability (Cronbach's alpha = .91) and construct validity in a sample of staff working in Korean long-term care facilities (Sagong et al., 2018). The Cronbach's alpha was .92 in this study.

#### Demographic and organizational characteristics

Demographic characteristics included age (in years), marital status (with or without spouse), educational level (high school or lower, college diploma, or bachelor's degree or higher), type of job (nursing staff or PCW), shift work (yes or no), and job tenure (number of months working at the current facility). Organizational characteristics included type of facility (long-term care hospital or nursing home), location (urban or rural), ownership (not-for-profit [incorporated] or for-profit), years in operation, number of beds, and staffing levels (resident-to-nursing staff ratio and resident-to-PCW ratio).

### Statistical Analysis

Descriptive statistics were used to summarize the characteristics of the staff and long-term care facilities. To examine the relationships between the person-centered care environment and the two staff outcomes (job satisfaction and turnover intention), multilevel analyses were used with Mplus Version 7.0 (Muthén & Muthén, Los Angeles, CA, USA), adjusted for the nested data structure, with individual care staff nested in their respective facilities. Specifically, two-level linear regression analysis was performed for job satisfaction, and two-level logistic regression analysis was performed for turnover intention. In these multilevel modeling analyses, the first level was individual care staff and the second level was long-term care facilities. Maximum likelihood estimation with robust standard errors was used to consider the nonnormal distribution of the two staff outcome variables.

## Results

### General Characteristics of the Long-Term Care Facility Sample

The characteristics of the 13 long-term care facilities included in this study are presented in Table 1. Of these facilities, nine were nursing homes and four were long-term care hospitals. Eleven were located in urban areas, and seven were for-profit facilities. The average number of years in operation was 10.9, the average resident-to-nursing staff ratio was 16.2, and the average resident-to-PCW ratio was 3.8.

**Table 1.** General Characteristics of the Long-Term Care Facilities Sample (*N* = 13)

Variable	Total (*N* = 13)		NH (*n* = 9)		LTCH (*n* = 4)	
	*n*	%	*n*	%	*n*	%
Location						
Urban	11	84.6	7	77.8	4	100.0
Rural	2	15.4	2	22.2	0	0.0
Ownership						
Not-for-profit (incorporate)	6	46.2	5	55.6	1	25.0
For-profit	7	53.8	4	44.4	3	75.0

Variable
	*M*	*SD*	*M*	*SD*	*M*	*SD*
Operating period (years)	10.9	6.0	12.2	6.7	7.8	2.2
Number of beds	111.7	81.4	72.8	38.3	199.3	88.3
Resident-to-nursing staff ratio	16.2	9.6	21.5	5.9	4.3	0.1
Resident-to-personal care worker ratio	3.8	2.3	2.7	0.4	6.2	3.1

***Note.*** NH = nursing home; LTCH = long-term care hospital.

### General Characteristics of the Care Staff Sample

In Table 2, the 235 staff participants included 94 nursing staff (RNs and CNAs) and 141 PCWs. The average age was approximately 52.9 years, and most (98.3%) were female. Almost two thirds of the respondents (56%) had a high school diploma or lower, and the average tenure at their current facility was 45.6 months (3.8 years). The mean scores for person-centered care environment and job satisfaction were 4.7 and 2.9, respectively. Almost 17.3% of the respondents reported that they intended to leave (turnover intention) within 1 year.

**Table 2.** General Characteristics of the Study Participants (*N* = 235)

Variable	Total		Job Satisfaction				Turnover Intention			
	*n*	%	*M*	*SD*	*t*/*F*	*p*	*n*	%	ϰ^2^	*p*
Age^a^ (years; *M* and *SD*)	52.9	9.4			2.711	.046			6.854	.077
21–40	27	11.7	2.8	0.5			9	33.3		
41–50	47	20.3	2.9	0.4			9	19.1		
51–60	114	49.4	2.9	0.4			14	12.3		
61–70	43	18.6	3.1	0.5			5	11.6		
Gender^a^					−1.688	.186			9.501	.002
Female	228	98.3	2.9	0.5			35	15.4		
Male	4	1.7	2.6	0.4			3	75.0		
Marital status^a^					0.813	.420			0.210	.646
With spouse	202	86.3	2.9	0.5			34	16.8		
Without spouse	32	13.7	3.0	0.4			4	12.5		
Educational level^a^					1.361	.258			6.075	.048
High school diploma or lower	131	56.0	3.0	0.5			14	10.7		
College diploma	54	23.1	2.8	0.4			11	20.4		
Bachelor's degree or higher	49	20.9	2.9	0.4			13	26.5		
Type of job					2.891	.004			2.950	.086
Nursing staff	94	40.0	2.8	0.4			21	22.3		
Personal care worker	141	60.0	3.0	0.5			17	12.1		
Shift work^a^					−0.099	.921			0.081	.776
Yes	84	36.1	2.9	0.5			23	27.4		
No	149	63.9	2.9	0.4			15	10.1		
Monthly income^a^ ($; *M* and *SD*)	1,635.0	341.4			0.843	.471			1.835	.607
< 1,500	93	40.3	3.0	0.5			13	14.0		
1,500–1,999	89	38.5	2.9	0.5			15	16.9		
2,000–2,499	43	18.6	2.8	0.4			9	20.9		
≥ 2,500	6	2.6	2.9	0.3			0	0.0		
Job tenure (months; *M* and *SD*)	45.6	38.4								
Person-centered care environment (*M* and *SD*)	4.7	0.6								
Job satisfaction (*M* and *SD*)	2.9	0.5								
Turnover intention (*n* and %)	38	16.2								

^a^Missing data.

### Results of Multilevel Regression Analyses

The results of multilevel regression analysis for job satisfaction and turnover intention are presented in Table 3. In the two-level linear regression model for job satisfaction, person-centered care environment was statistically associated with job satisfaction. For each one-unit increase in person-centered care environment, job satisfaction among care staff increased 0.49 points, on average. For other individual care staff and organizational characteristics, facility type (long-term care hospitals compared with nursing homes) and the resident–nursing staff ratio were found to be statistically and negatively associated with job satisfaction.

**Table 3.** Results of Hierarchical Linear/Logistic Regression Analysis

Variable	Job Satisfaction (*n* = 230)			Turnover Intention (*n* = 220)	
	β	*SE*	*p*	*OR*	CI
Job satisfaction	─	─	─	0.847	[0.739, 0.971]
Person-centered care environment	0.49	0.05	<.001	0.890	[0.796, 0.995]
Individual factors					
Age (years)	0.13	0.07	.070	0.877	[0.754, 1.020]
Educational level (ref: high school or lower)					
College diploma	0.04	0.09	.664	1.082	[0.932, 1.255]
Bachelor's degree or higher	−0.01	0.07	.848	1.157	[1.029, 1.301]
Monthly income ($)	−0.04	0.07	.525	0.931	[0.822, 1.053]
Nursing staff (ref: personal care worker)	−0.11	0.06	.072	1.066	[0.921, 1.234]
Shift work (ref: fixed work)	−0.09	0.05	.069	1.089	[0.940, 1.261]
Job tenure (months)	0.04	0.06	.496	0.953	[0.850, 1.068]
Organizational factors					
Long-term care hospital (ref: nursing home)	−0.62	0.25	.012	2.192	[1.315, 3.655]
Urban location (ref: rural)	0.20	0.14	.157	0.638	[0.459, 0.884]
For-profit (ref: not-for-profit)	−0.02	0.09	.802	1.005	[0.946, 1.066]
Number of beds	0.32	0.22	.144	1.110	[0.827, 1.489]
Resident-to-nursing staff ratio	−0.67	0.21	.001	2.199	[1.339, 3.615]
Resident-to-personal care worker ratio	−0.21	0.21	.310	0.837	[0.620, 1.130]

***Note.*** β = standardized coefficient; *SE* = standard error; *OR* = odds ratio; CI = confidence interval; ref = reference.

In the two-level logistic regression model for turnover intention, which included both person-centered care environment and job satisfaction, person-centered care environment was statistically associated with turnover. For each one-unit increase in person-centered care environment, the care staff participants were 11% less likely to respond affirmatively to the turnover intention question. In addition, job satisfaction was statistically and negatively associated with turnover intention. Among individual staff, those holding a bachelor's degree or higher were more likely to respond affirmatively to the turnover intention question than those holding a high school diploma or lower. With regard to organizational characteristics, turnover intention was statistically higher in long-term care hospitals, in facilities located in a rural area, and in facilities with higher resident-to-nursing staff ratios.

## Discussion

This study was conducted to investigate the relationship between person-centered care environment and, respectively, job satisfaction and turnover intention among RNs, CNAs, and PCWs working in long-term care facilities in Korea. The findings of this study indicate a significant association between person-centered care environments and job satisfaction and turnover intention among nursing staff after adjusting for individual and organizational characteristics such as age, educational level, ownership type, and staffing levels. Consistent with the results of a previous study on the development of person-centered care environments in nursing homes (McCormack et al., 2010), this study revealed that increasing the person-centric nature of care environments contributes significantly to higher job satisfaction among long-term care staff. Moreover, this study was the first to provide empirical evidence regarding the importance of person-centered care environments to the improvement of staff outcomes in Korean long-term care facilities such as increased job satisfaction and decreased turnover intention.

As regards the underlying mechanism, a prior study used mediation analysis to explore how person-centered care environments influence positive staff outcomes, including job satisfaction and burnout (te Boekhorst et al., 2008), finding that in small-scale living homes, care staff felt they had more work control, perceived fewer job demands, and received more social support from their coworkers. Reducing job demands in a care setting increases the likelihood that care staff support colleagues and experience less emotional exhaustion. Moreover, staff empowerment is an important component of person-centered care (Koren, 2010). Nursing staff who work in person-centered care environments are more likely to control their work and actively participate in care practices, which increases job satisfaction (Rokstad et al., 2015).

To enhance job satisfaction among nursing staff, who play a vital role in providing better resident care (McGilton et al., 2016), long-term care facility administrators should consider implementing person-centered care. In this study, the reported average score for person-centered care environments was slightly lower than the score from a previous study that applied the same PCQ-S (Edvardsson et al., 2009) to nurse assistants working in residential care settings in southern Sweden (Wallin et al., 2012). Although much attention has been given to the person-centered care model, and some nursing home administrators have attempted to develop a person-centered culture in Korea (M. Lee et al., 2013), much work remains ahead to create a care environment that provides individualized and person-centered care to accommodate the unique needs, preferences, and dignity of each resident in the very limited number of long-term care facilities in Korea.

Turnover intention was examined in a separate regression model that included both person-centered care environment and job satisfaction as potential predictors. As expected, a statistically significant relationship was found between person-centered care environment and turnover intention. In the regression model, there was a significant association between job satisfaction and turnover intention among nursing staff, which corroborates the findings of previous studies, indicating a strong link between job satisfaction and turnover intention among long-term care staff (Choi & Johantgen, 2012; Karantzas et al., 2012; Kuo et al., 2014). As was shown by Kuo et al. (2014), job satisfaction had a significant, mediating effect on the relationship between work stress and turnover intention (Kuo et al., 2014). The question of whether job satisfaction mediates the relationship between person-centered care environment and turnover intention is beyond the scope of this study. Given that our findings showed a positive association between person-centered care environment and job satisfaction and, in a separate regression model, a significant association between this environment and turnover intention, further research is needed to explore the comprehensive relationships among person-centered care environment, job satisfaction, and turnover intention among long-term care staff.

With regard to care staff characteristics, age and educational level were found to be significantly and positively related, respectively, to job satisfaction and turnover intention. These results are consistent with the findings of a previous study on job satisfaction and turnover intention among CNAs working in nursing homes in the United States (Choi & Johantgen, 2012). Particularly in Korea, although these two demographic characteristics are the most basic qualifications when hiring staff in long-term care settings, facility administrators must sensibly consider these characteristics in addition to personal needs and motivators during employment interviews.

With regard to organizational characteristics, as expected, on the basis of results of previous studies (Donoghue, 2010; Karantzas et al., 2012; Zhang et al., 2019), a significant association was found between staffing level and job satisfaction as well as turnover intention. Specifically, the resident-to-nursing staff ratio was significantly associated with job satisfaction as well as turnover intention in this study. In addition, location was significantly related to turnover intention but not to job satisfaction. In this study, nursing staff working in urban long-term care facilities were less likely than their counterparts in rural settings to report turnover intention. This result may be related to the relatively poorer quality of care and working conditions often experienced in long-term care facilities in rural areas in Korea (B. R. Lee, 2016).

### Limitations

This study may have been affected by several limitations. Data were gathered from a convenience sample of nursing staff working in 13 long-term care facilities. In addition, the sample size was relatively small for multilevel regression analysis, which may reduce its statistical power, as it is usually recommended to have as many observations as possible at the top level of the multilevel model (Snijders, 2005). Thus, the results should be interpreted with caution and may not be generalizable to staff working in long-term care facilities throughout Korea. Moreover, we examined person-centered care environment and other factors related to job satisfaction and turnover intention using data from all care staff who were involved in direct resident care, including RNs, CNAs, and PCWs. Although CNAs are considered nursing staff in Korean long-term care facilities, CNAs and PCWs are usually considered paraprofessional nursing personnel and are likely to provide most direct resident care under the supervision of RNs (Choi & Johantgen, 2012; McGilton et al., 2016). Some studies have found that supportive supervision is one of the most significant factors related to turnover intention among PCWs (Bethell et al., 2018; Radford et al., 2015). Perceptions of person-centered care environments as well as factors influencing job satisfaction and turnover intention among RNs may differ from CNAs and PCWs. Thus, more research is needed to better understand the influence of person-centered care environments and other factors on job satisfaction and turnover intention at each staffing level in long-term care facilities.

### Conclusions

Despite the limitations, the findings of this study indicate that promoting a person-centered care environment in long-term care facilities is vital to increasing job satisfaction and decreasing turnover intention in care staff who work in these facilities. In the long-term care practice, closer relationships and improved communications between staff and residents may decrease staff burnout and work-related emotional exhaustion and increase job satisfaction. Furthermore, care staff who provide individualized care to residents based on person-centeredness are more likely to experience enhanced confidence, which may suggest a significant strategy for retaining competent care staff in long-term care settings. Thus, more efforts should be made to develop person-centered care environments that facilitate tailored care processes that address the unique value, dignity, and preferences of individuals through a therapeutic relationship between all care providers and residents. Related changes will benefit not only residents but also care staff, who are invaluable caregivers in long-term care facilities in Korea.
